# Stochastic Learning in Kolkata Paise Restaurant Problem: Classical and Quantum Strategies

**DOI:** 10.3389/frai.2022.874061

**Published:** 2022-05-26

**Authors:** Bikas K. Chakrabarti, Atanu Rajak, Antika Sinha

**Affiliations:** ^1^Condensed Matter Physics Division, Saha Institute of Nuclear Physics, Kolkata, India; ^2^S. N. Bose National Centre for Basic Sciences, Kolkata, India; ^3^Economic Research Unit, Indian Statistical Institute, Kolkata, India; ^4^Department of Physics, Presidency University, Kolkata, India; ^5^Department of Computer Science, Asutosh College, Kolkata, India

**Keywords:** collective learning, critical slowing down, decoherence, KPR problem, minority game, quantum entanglement, three-player quantum KPR

## Abstract

We review the results for stochastic learning strategies, both classical (one-shot and iterative) and quantum (one-shot only), for optimizing the available many-choice resources among a large number of competing agents, developed over the last decade in the context of the Kolkata Paise Restaurant (KPR) Problem. Apart from few rigorous and approximate analytical results, both for classical and quantum strategies, most of the interesting results on the phase transition behavior (obtained so far for the classical model) uses classical Monte Carlo simulations. All these including the applications to computer science [job or resource allotments in Internet-of-Things (IoT)], transport engineering (online vehicle hire problems), operation research (optimizing efforts for delegated search problem, efficient solution of Traveling Salesman problem) will be discussed.

## 1. Introduction

Game theory was initially developed to investigate different strategic situations with competing players (Morgenstern and Von Neumann, 1953). Of late, the concept of game theory is being applied to different statistical events to measure the success rate when one's success depends on the choice of the other agents. The game of Prisoners' dilemma (refer to e.g., Prisoner's Dilemma, 2019) is a popular example where two non-communicating (or non-interacting) agents choose their actions from two possible choices. It is a two-person, two-choice, one-shot (one-time decision) game. The Nash equilibrium (refer to e.g., Osborne and Rubinstein, 1994) solution employs the strategy, where the other player can not gain from any of the choices, and both the players necessarily defect. However, this is not a Pareto optimal solution (refer to e.g., Lockwood, 2008), where no change in the decision can lead to a gain for one player without any loss for the other. This problem has been used to model many real life problems such as auction bidding, arms races, oligopoly pricing, political bargaining, and salesman effort.

The minority game theory (refer to e.g., Challet et al., 2005) generalizes this idea of a very large number of non-communicating players with two choices for each of them. As the name suggests, the players who make the minority group choice (at any time) receive a payoff. This game is not a one-shot game, and the players learn from their previous mistakes (loss of payoffs) and continuously try to upgrade their respective strategies to gain the payoffs and they (the society as a whole) learn collectively to reach a level of maximum efficiency, where no one can improve their payoff any further. A phase transition (refer to e.g., Challet et al., 2005) occurs at a critical value of the memory size (number of distinct strategies individually remembered; assumed to be the same for all the players) and the number of players and the socially optimal learning time diverges at this critical point (refer to e.g., Stanley, 1987). The game has many important applications of social dilemmas, including a decision of making an investment in a stock market; over-crowding of the agents any day due to the decision of either buying or selling a particular stock in the financial market can lead to a loss for the majority of players.

The minority game is further generalized for many choices in addition to many players (as the minority game) in the Kolkata Paise Restaurant (KPR) game theory, introduced by Chakrabarti (2007) and Chakrabarti et al. (2009) (for a recent review refer to e.g., Chakrabarti et al., 2017). The KPR game is also an iterative game, played by the agents or players without any interaction or communication between each other.

Long ago in Kolkata, there were very cheap and fixed price “Paise Restaurants” (also called “Paise Hotels”; Paise was the smallest Indian coin) which were very popular among the daily laborers in the city. During lunch hours, these laborers used to walk down (to save the transport costs) from their place of work to one of these restaurants. These Paise Restaurants would prepare everyday a fixed (small) number of such dishes, and if several groups of laborers would arrive any day at the same restaurant, only one group perhaps would get their lunch and the rest would miss lunch that day. There were no cheap communication means (mobile phones) for mutual interactions, in order to decide about the respective restaurants of the day. Walking down to the next restaurant would mean failing to report back to work on time. To complicate this collective learning and decision making problem, there were indeed some well-known rankings of these restaurants, as some of them would offer tastier items compared to the others (at the same cost, paisa, of course) and people would prefer to choose the higher rank of the restaurant, if not crowded. This “mismatch” of the choice and the consequent decision not only creates inconvenience for the prospective customer (going without lunch), but would also mean “social wastage” (excess unconsumed food, services, or supplies somewhere).

A similar problem arises when the public administration plans and provides hospitals (beds) in different localities, but the local patients prefer “better” perceived hospitals elsewhere. These “outsider” patients then compete with the local patients and have to choose other suitable hospitals elsewhere. Unavailability of the hospital beds in the overcrowded hospitals may be considered as insufficient service provided by the administration, and consequently, the unattended potential services will be considered a social wastage. Playing this kind of game, anticipating the possible strategies of the other players and acting accordingly, is very common in society. Here, the number of choices need not be very limited (as in the standard binary-choice formulations of most of the games), and the number of players can be truly large. Also, these are not necessarily one-shot games, rather the players can learn from past mistakes and improve on the selection of their strategies for the next move. These features make the games extremely intriguing and also versatile, with major collective or socially emerging structures.

The KPR problem seems to have a trivial solution: suppose that somebody, say a dictator (who is not a player), assigns a restaurant to each person and asks them to shift to the next restaurant cyclically, on successive evenings. The fairest and most efficient solution: each customer gets food each evening (if the number of plates or choices is the same as that of the customers or players) with the same share of the rankings as others, and that too from the first evening (minimum evolution time). This, however, is not a true solution to the KPR problem, where each customer or agent decides on their own every evening, based on complete information about past events. Several recent applications of the classical KPR strategies to the Vehicle for Online Hire problem (Martin, 2017; Martin and Karaenke, 2017), resource allocation problem in the context of Internet-of-Things (IoT) (Park and Saad, 2017), development of a different strategy for solving the Traveling Salesman Problem (Kastampolidou et al., 2021), etc have been made.

In recent decades, quantum game theory has been developed, promising more success than classical strategies (Eisert et al., 1999; Meyer, 1999; Marinatto and Weber, 2000; Benjamin and Hayden, 2001; Piotrowski and Sładkowski, 2003; Bleiler, 2008; Salimi and Soltanzadeh, 2009; Landsburg, 2011). This is an interdisciplinary approach that connects three different fields: quantum mechanics, information theory, and game theory in a concrete way. Quantum game theory offers different protocols that are based on the uses of quantum mechanical phenomena like entanglement, quantum superposition, and interference arising due to wave mechanical aspects of such systems. In the context of game theory, quantum strategies are first introduced in two articles by Eisert et al. (1999) and Meyer (1999) where they showed that a player performing a quantum move wins against a player performing a classical move regardless of their classical choices. The advantage of a quantum strategy over a classical one has been specifically investigated in Eisert et al. (1999) for the case of Prisoners' dilemma. This idea is generalized for multiple players by Benjamin and Hayden (2001) with a specific solution for four players. The authors here introduced the quantum minority game where they showed that an entanglement shared between the players promises better performance of quantum strategy over the classical one. Chen et al. (2004) further extends this result of quantum minority game for *N*-players.

Since then, different aspects of multi-player quantum minority games are being studied extensively. As already mentioned, the KPR problem is a minority game with a large number of choices for each of the players, who are also equally large in number. Sharif and Heydari (2011) introduced the quantum version of the KPR game, with a solution for three agents and three choices. This study was later extended by Sharif and Heydari (2012a), Ramzan (2013), and Sharif and Heydari (2013) for the three and multi-player quantum minority games, including the quantum KPR games (essentially one-shot solutions). For a detailed discussion refer to Chakrabarti et al. (2017).

We review here the statistics of the KPR problem employing both classical and quantum strategies. The article is organized as follows. In Section 2, we describe the classical strategies of the KPR game and show that there exists a phase transition when the number of customers is less than the number of restaurants. We also discuss there the possible ways by which we can minimize the social wastage fraction. In Section 3, we first discuss the general setting of quantum games and then provides a flavor of two game theoretical problems, such as Prisoners' dilemma and minority game in the context of both classical and quantum strategies. In Section 4, we introduce the quantum version of the KPR problem. We review here the results of the one-shot quantum KPR problem with three players and three choices by Sharif and Heydari (2011, 2012a,b), and Ramzan (2013). We show that by using quantum strategies one can gain in payoff by 50% compared to the classical strategies for a one-shot KPR game with three players and three choices. We also discuss the effect of entanglement and decoherence (or loss of phase coherence) in finding the expected payoff of a player for the above mentioned problem.

## 2. Statistics of KPR Game: Classical Strategies

Let us consider the KPR game with *N* restaurants and λ*N* non-communicating players (agents or customers). We assume that everyday or evening or time (*t*), each restaurant prepares only one dish (generalization to a larger number would not affect the statistics of the game). As discussed, every time *t*, the objective of each of the players is to choose one among *N* restaurants such that she will be alone there in order to get the only dish. If some restaurant is visited by more than one customer, then the restaurant selects one of them randomly and serves the dish to her; thus, the rest of the visitors there would remain unhappy by starving that evening.

Let us consider first the random choice (no learning) case where each player chooses randomly any of the restaurants. Then the probability *P* of choosing one restaurant by *n* (≤ *N*) players is

(1) $$\begin{array}{l} P\left(n\right)=\left(\begin{array}{c} \text{λ}N\\ n \end{array}\right)p^{n}{\left(1-p\right)}^{\lambda N-n};p=\frac{1}{N}. \end{array}$$

In the case of *N* going to infinity, we get

(2) $$\begin{array}{l} P\left(n\right)=\frac{\lambda^{n}}{n!}exp\left(-\lambda \right). \end{array}$$

Hence, the fraction *P*(*n* = 0) of restaurants not chosen by any customer is *exp*(−λ). The fraction of restaurants chosen by at least one customer on any evening is, therefore, the utilization fraction (Chakrabarti et al., 2009)

(3) $$\begin{array}{l} f=1-exp\left(-\lambda \right). \end{array}$$

If *N* agents (where λ = 1) randomly choose and visit anyone among *N* restaurants then utilization fraction *f* becomes 1 − *exp*(−1) ≃ 0.63. Since there is no iterative learning for this case, every time the utilization fraction will be about 63% starting from the first day (convergence time τ = 0).

It may be noted that a dictated solution to the KPR problem is simple and very efficient from the first day. The Dictator is not a player in the game and asks the players to form a queue (with periodic boundary conditions), visit a restaurant according to her respective positions in that queue, and continue shifting by one step every day. Every player gets a dish, and hence, the steady state (*t*-independent) social utilization fraction *f* becomes maximum (unity) from the first day (τ = 1). This dictated solution is applicable even when the restaurants have ranks (agreed by all the customers) i.e., agents have their preferences over the restaurants. Thus, the dictated solution is very efficient in achieving maximum utility from the first day (*f* = 1, τ = 1). However, no choice of the individual is considered here and in a democratic set-up no such a dictatorial strategy is acceptable.

We now consider the case where the players try to learn and update their strategies for choosing a restaurant to avoid overcrowding the chosen restaurant. As already discussed, we measure the social utilization fraction *f*(*t*) on any day *t* as

(4) $$\begin{array}{l} f\left(t\right)=\overset{N}{\underset{i=1}{\sum }}\left[\delta \left(n_{i}\left(t\right)\right)/\lambda N\right], \end{array}$$

where δ(*n*) = 1 for *n* ≥ 1 and δ(*n*) = 0 for *n* = 0; *n*_*i*_(*t*) denotes the number of customers arriving at the *i*th (rank if customer choice is considered) restaurant on *t* th evening. The goal is to learn collectively toward achieving *f*(*t*) = 1 preferably in finite convergence time τ, i.e., *f*(*t*) = 1 for *t* ≥ τ, where τ is finite.

Earlier studies (refer to e.g., Chakrabarti et al., 2009, 2017; Ghosh et al., 2010a,b, 2012; Sinha and Chakrabarti, 2020) had proposed several learning strategies for the KPR game. In Ghosh et al. (2010b), Ghosh et al. (2012), Sinha and Chakrabarti (2020), and Chakrabarti and Sinha (2021), the authors had studied several stochastic crowd avoidance learning strategies leading to increased utilization fraction (compared to the random choice case Equation 3). In some of the cases, this is achieved (*f* = 1) at a critical point (Stanley, 1987) where τ goes to infinity due to critical slowing down.

Here, we discuss numerical (Monte Carlo) results for the statistics of the KPR game where λ*N* (λ > 0) customers choose one among *N* restaurants following a strategy discussed next. On day *t*, an agent goes back to her last day's visited restaurant *k* with a probability

(5a) $$\begin{array}{l} p_{k}\left(t\right)={\left[n_{k}\left(t-1\right)\right]}^{-\alpha };\;\alpha >0 \end{array}$$

and chooses a different restaurant (*k*′ ≠ *k*) among any of the (*N* − 1) neighboring restaurants, with probability

(5b) $$\begin{array}{l} p_{k^{\prime }}\left(t\right)=\left(1-p_{k}\left(t\right)\right)/\left(N-1\right). \end{array}$$

These “learning” strategies employed by the players, for the choice of restaurants placed on different dimensional (*d*) lattices. In infinite dimension (mean field case), the restaurant indices *k* and *k*′ in Equation (5a, 5b) run from 1 to *N* of the lattice. For finite dimensions, *k* runs from 1 to *N* while *k*′ corresponds to the nearest neighbor of the *k*th restaurant on the lattice.

Authors in Ghosh et al. (2012) had studied crowd dynamics with α = 1, λ = 1 in infinite, 2*d*, 1*d* lattice structure of restaurants. KPR dynamics for α ≤ 1, λ = 1 had been studied in Sinha and Chakrabarti (2020) for infinite, 3*d*, 2*d*, 1*d* lattice structure of restaurants. Phase transition behaviors are observed for α near α_*c*_ = 0_+_ for infinite, 3*d* and 2*d* lattice structure of the restaurants. The steady state statistics are studied when the utilization fraction *f*(*t*) remains the same (within a predefined margin) for further iterations. The steady state wastage fraction (1 − *f*) and the convergence time τ for reaching the steady state are found to vary with Δα ≡ |α − α_*c*_| as (1 − *f*) ~ Δα^β^ and τ ~ Δα^−γ^ with β ≃ 0.8, 0.87, 1.0 and γ ≃ 1.18, 1.11, 1.05 in infinite-dimension, 3*d* and 2*d* lattice structures, respectively. Results of 1*d* lattice structure are found to be trivial unlike other dimensions and no phase transition (*f* reaches unity with no divergence in τ) is seen for any α > 0.

**Figure 1:**
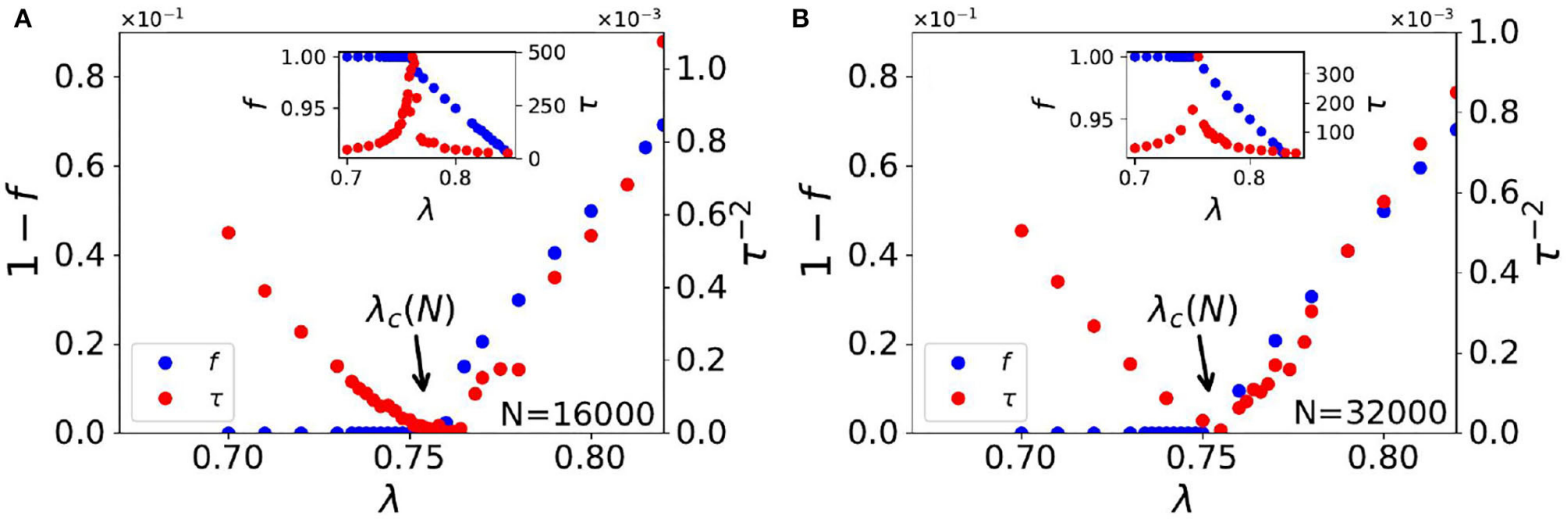
Plots of steady state convergence behavior in infinite dimensional lattice; Social wastage fraction (1 − *f*) and convergence time τ against customer fraction λ. Observed power laws are (1 − *f*) ~ Δλ^β^ where β = 1.0 ± 0.02 and τ ~ Δλ^−γ^ where γ = 0.5 ± 0.02. Here, Δλ ≡ |λ − λ_*c*_(*N*)|. The insets show the variation of *f* and τ against λ, showing the diverging behavior of τ where *f* reaches unity at λ = λ_*c*_(*N*). **(A)** for *N* = 16,000 and **(B)** for *N* = 32,000.

**Figure 2:**
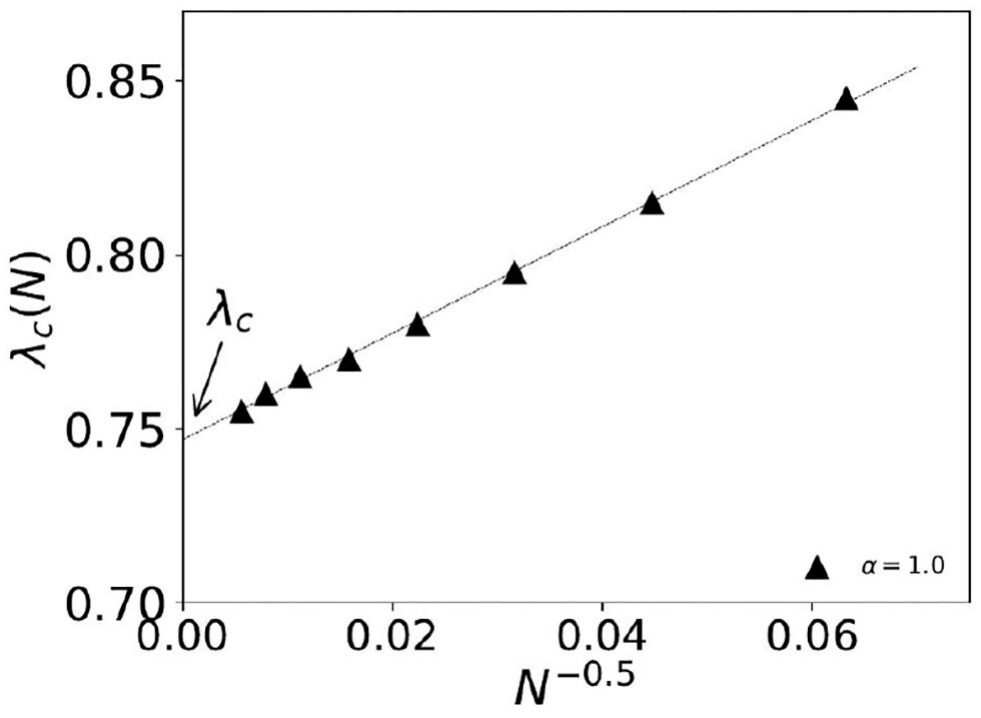
Extrapolation study of the effective finite size dependent critical density of customers λ_*c*_(*N*). The system size dependence is fitted to $\frac{1}{\sqrt{N}}$ and we get λ_*c*_ = 0.74 ± 0.01 for λ_*c*_ ≡ λ_*c*_(*N* → ∞).

Here, we discuss the numerical results of the Monte Carlo studies on steady state statistics of the KPR game dynamics for general α and λ cases. In the case where α = 1 and λ is < 1, we find power law fits for social wastage fraction (1 − *f*) ~ Δλ^β^ and convergence time τ ~ Δλ^−γ^ with β = 1.0 ± 0.02 and γ = 0.5 ± 0.02 in infinite dimension with Δλ ≡ |λ − λ_*c*_|, where λ_*c*_ = 0.74 ± 0.01 (refer to Figures 1, 2). For finite size *N*, we observe the effective critical point λ_*c*_(*N*) for which the finite size scaling (refer to Figure 2) gives the best fit for *dν* = 2.0 and λ_*c*_ = 0.74 ± 0.01.

A crude estimate of the mean field value of λ_*c*_ can be obtained as follows. Here, λ*N* agents choose every day among the *N* restaurants. Hence, the probability of any restaurant being chosen by a player is $\frac{\lambda N}{N}=\lambda$, and the fraction of restaurants not visited by any player will be (1 − λ). In the steady state, the number of players *n* choosing any restaurant can be 0, 1, 2, 3, …. If we assume the maximum crowd size at any restaurant on any day to be 2, then the probability of those restaurants going vacant next day will be ${\left(\frac{1}{2}\right)}^{2}$. Hence, the critical value λ_*c*_ of λ in the steady state will be given by

(6) $$\begin{array}{l} 1-\lambda_{c}=\frac{1}{4}, \end{array}$$

giving λ_*c*_ ≃ 0.75. For more details refer to Ghosh et al. (2012).

## 3. Quantum Games

In the setting of quantum games, the *N* different choices of any arbitrary player or agent are encoded in the basis states of an *N*-level quantum system that acts as a subsystem with *N*-dimensional Hilbert space. The total system for *M* (= λ*N* as defined in Section 2) players or agents can be represented by a state vector in a $\prod_{i=1}^{M}\text{dim}\left(H_{L_{i}}\right)$ dimensional Hilbert space $H_{L}=H_{L_{M}}⊗H_{L_{n-1}}\cdots ⊗H_{L_{1}}$, where $H_{L_{i}}$ is the Hilbert space of the *i*-th subsystem. The different subsystems are distributed among the players and the initial state of the total system is chosen so that the subsystems become entangled. The players do not communicate with each other before choosing a strategy. A strategy move in quantum games is executed by the application of local operators associated with each player on the quantum state. The players do not have access to any other parts of the system except their own subsystems. In addition, no information is shared between the players exploiting the quantum nature of the game. The quantum strategies are indeed the generalized form of classical strategies with *s*_*i*_ ∈ *S*_*i*_ ⇒ *U*_*i*_ ∈ S(*N*_*i*_), where the set of permitted local quantum operations S(*N*_*i*_) is some subset of the special unitary group SU(*N*_*i*_).

We will now describe different steps of the quantum game protocol (Sharif and Heydari, 2012b). The game starts with an initially entangled state |ψ_*n*_〉 shared by different players. We have considered the subsystems of the same dimension *N* that indeed denotes the number of pure strategies available to each player. The number of subsystems is equal to the number of players. It can be thought that |ψ_*n*_〉 has been prepared by a referee who distributes the subsystems among the players. By choosing a unitary operator *U* from a subset of SU(*N*), the players apply that on their subsystems and, the final state is given by

(7) $$\begin{array}{l} \rho_{\text{fin}}=U⊗U⊗\cdots ⊗U\rho_{\text{in}}U^{†}⊗U^{†}⊗\cdots ⊗U^{†}, \end{array}$$

where ρ_in_ and ρ_fin_ are the initial and final density matrix of the system, respectively. Due to the symmetry of the games and, since the players do not communicate among themselves, they are supposed to do the same operation. The advantage of the quantum game over the classical one is that it reduces the probability of collapsing the final state ρ_fin_ to the basis states that have lower or zero payoff $. Since quantum mechanics is a fundamentally probabilistic theory, the only notion of payoff after a strategic move is the expected payoff. To evaluate the expected payoffs, the first step is to define a payoff operator *P*_*i*_ for an arbitrary player *i* and that can be written as

(8) $$\begin{array}{l} P_{i}=\underset{j}{\sum }{\$}_{i}^{j}|\alpha_{i}^{j}\rangle \langle \alpha_{i}^{j}|, \end{array}$$

where ${\$}_{i}^{j}$ are the associated payoffs to the states $|\alpha_{i}^{j}\rangle$ for *i*-th player. The expected payoff *E*_*i*_($) of player *i* is then calculated by considering the trace of the product of the final state ρ_fin_ and the payoff-operator *P*_*i*_,

(9) $$\begin{array}{l} E_{i}\left(\$\right)=Tr\left(P_{i}\rho_{fin}\right). \end{array}$$

The Prisoners' dilemma is a game with two players and both of them have two independent choices. In this game, two players, Alice and Bob choose to cooperate or defect without sharing any prior information about their actions. Depending on their combination of strategies, each player receives a particular payoff. Once Bob decides to cooperate, Alice receives payoff $_*A*_ = 3 if she also decides to cooperate, and she receives $_*A*_ = 5 if she decides to defect. On the other hand, if Bob sets his mind to defect, Alice receives $_*A*_ = 1 by following Bob and, $_*A*_ = 0 by making the other choice. It reflects that whatever Bob decides to choose, Alice will always gain if she decides to defect. Since there is no possibility of communication between the players, the same is true for Bob. This leads to a dominant strategy when both the players defect and they both have a payoff of $_A/B_ = 1. In terms of game theory, this strategy of mutual defection is a Nash equilibrium, because none of the players can do better by changing their choices independently. However, it can be noted that this is not an efficient solution. Because there exists a Pareto optimal strategy when both the players cooperate, and they both receive $_A/B_ = 1. This gives rise to a dilemma in this game. After a few decades, the quantum version of this game is introduced by Eisert et al. (1999). In the quantum formulation, the possible outcomes of classical strategies (cooperate and defect) are represented by two basis vectors of a two-state system, i.e., a qubit. For this game, the initial state is considered as a maximally entangled Bell-type state, and the strategic moves for both the players are performed by the unitary operators from the subset of the SU(2) group. In this scenario, a new Nash equilibrium is emerged in addition to the classical one, i.e., when both the players choose to defect. For the new case, the expected payoffs for both the players are found to be *E*($_*A*_) = *E*($_*B*_) = 3. This is exactly the Pareto optimal solution for the classical pure strategy case. In the quantum domain, this also becomes a Nash equilibrium. Thus, considering a particular quantum strategy one can always get an advantage over a classical strategy.

The above game is generalized for multiple players with two choices in the minority game theory. In this game, *n* non-communicating agents independently make their actions from two available choices, and the main target of the players is to avoid the crowd. The choices are then compared and the players who belong to the smaller group are rewarded with a payoff $ = 1. If two choices are evenly distributed, or all the players make the same choice, no player will get any reward. To get the Nash equilibrium solution, the players must choose their moves randomly, since the deterministic strategy will lead to an undesired outcome where all the players go for the same choice. In this game, the expected payoff *E*($) for a player can be calculated by the ratio of the number of outcomes that the player is in the minority group and the number of different possible outcomes. For a four-player game, the expected payoff of a player is found to be 1/8, since each player has two minority outcomes out of sixteen possible outcomes. A quantum version of this game for four players was first introduced by Benjamin and Hayden (2001). They have shown that the quantum strategy provides better performance than the classical one for this game. The application of quantum strategy reduces the probability of even distribution of the players between two choices, and this fact is indeed responsible for the outer performance of quantum strategy over classical one. The quantum strategy provides an expected payoff *E*($) = 1/4 for the four-player game which is twice of the classical payoff.

## 4. Quantum KPR Problem

As already mentioned, the Kolkata restaurant problem is a generalization of the minority game, where λ*N* non-communicating agents or players generally have *N* choices. The classical version of the KPR problem has been discussed in Section 2. This problem is also studied in the quantum mechanical scenario, where the players are represented by different subsystems, and the basis states of the subsystems are different choices. To remind, for the KPR game, each of λ*N* customers chooses a restaurant for getting the lunch from *N* different choices in parallel decision mode. The players (customers) receive a payoff if their choice is not too crowded, i.e., the number of customers with the same choices is under some threshold limit. For this problem, this limit is considered as one. If more than one customer arrives at any restaurant for their lunch, then one of them is randomly chosen to provide the service, and the others will not get lunch on that day.

Let us consider a simple case of three players, say, Alice, Bob, and Charlie who have three possible choices: restaurant 1, restaurant 2, and restaurant 3. They receive a payoff $ = 1 if they make a unique choice, otherwise they receive a payoff $ = 0. Therefore, it is a one-shot game, i.e., non-iterative, and the players do not have any knowledge from previous rounds to decide their actions. Since the players can not communicate, there is no other way except to randomize the choices. In this case, there is a total of 27 different combinations of choices and 12 of that provide a payoff $ = 1 to each of the players. Therefore, randomization between the choices leads to an expected payoff *E*_*c*_($) = 4/9 for each of the players using the classical strategy.

The quantum version of the KPR problem with three players (*M* = 3; λ = 1) and three choices (*N* = 3) is first introduced by Sharif and Heydari (2011). In this case, Alice, Bob, and Charlie share a quantum resource. Each of these players has a part in a multipartite quantum state. Whereas, the classical players are allowed to randomize between their discrete set of choices, for the quantum version, each subsystem is allowed to be transformed by local quantum operations. Therefore, choosing a strategy of choice is equivalent to choosing a unitary operator *U*. In absence of the entanglement in the initial state, it has been found that quantum games yield the same payoffs as its classical counterpart. On the other hand, it has been shown that sometimes a combination of unitary operators and entanglement outperform the classical randomization strategy.

In this particular KPR problem, the players have three choices, therefore, we need to deal with qutrits instead of qubits that are used for two choices to apply quantum protocols (Sharif and Heydari, 2012b). The local quantum operations on qutrits are performed by a complicated group of matrices from SU(3) group, unlike in the case of qubits where the local operators belong to the SU(2) group. A qutrit is a three-level quantum system on a three-dimensional Hilbert space $H_{L}={\text{C}}^{3}$. The most general form of the quantum state of a qutrit on the computational basis is given by

(10) $$\begin{array}{l} |\psi \rangle =c_{0}|0\rangle +c_{1}|1\rangle +c_{2}|2\rangle , \end{array}$$

where *c*_0_, *c*_1_, and *c*_2_ are three complex numbers satisfying the relation $|c_{0}{|}^{2}+|c_{1}{|}^{2}+|c_{2}{|}^{2}=1$. The basis states follow the orthonormal condition 〈*i*|*j*〉 = δ_*i,j*_, where *i, j* = 0, 1, 2. Then, the general state of an *n*-qutrit system can be written as a linear combination of 3^*n*^ orthonormal basis vectors:

(11) $$\begin{array}{l} |\text{Ψ}\rangle =\overset{2}{\underset{y_{n},..,y_{1}=0}{\sum }}c_{y_{n}...y_{1}}|y_{n}\cdots y_{1}\rangle , \end{array}$$

where the basis vectors are the tensor product of individual qutrit states, defined as,

(12) $$\begin{array}{l} |y_{n}\cdots y_{1}\rangle =|y_{n}\rangle ⊗|y_{n-1}\rangle ⊗\cdots ⊗|y_{1}\rangle \in H_{L}=\overset{n\text{-times}}{\overset{︷}{{\text{C}}^{3}⊗...⊗{\text{C}}^{3}}}, \end{array}$$

with *y*_*i*_ ∈ {0, 1, 2}. The complex coefficients satisfy the normalization condition $\sum |c_{y_{n}...y_{1}}{|}^{2}=1$.

A single qutrit can be transformed by a unitary operator *U* that belongs to the special unitary group of degree 3, denoted by SU(3). In a system of *n* qutrits, when an operation is performed only on a single qutrit, it is said to be local. The corresponding operation changes the state of that particular qutrit only. Under local operations, the state vector of a muti-qutrit system is transformed by the tensor products of individual operators, and the final state is given by

(13) $$\begin{array}{l} |{\text{Ψ}}_{\text{fin}}\rangle =U_{n}⊗U_{n-1}⊗\cdots ⊗U_{1}|{\text{Ψ}}_{\text{in}}\rangle , \end{array}$$

where |Ψ_in_〉 is the initial state of the system.

The SU(3) matrices, i.e., 3 × 3 unitary matrices are parameterized by defining three orthogonal complex unit vectors $\bar{u},\bar{v},\bar{w}$, such that $\bar{u}\cdot \bar{v}=0$ and ${\bar{u}}^{*}\times \bar{v}=\bar{w}$ (Mathur and Sen, 2001). A general complex vector with a unit norm is given by

(14) $$\begin{array}{l} \bar{u}=\left(\begin{array}{c} \sin\theta \cos\phi e^{i\alpha_{1}}\\ \sin\theta \sin\phi e^{i\alpha_{2}}\\ \cos\theta e^{i\alpha_{3}} \end{array}\right), \end{array}$$

where 0 ≤ ϕ, θ ≤ π/2, and 0 ≤ α_1_, α_2_, α_3_ ≤ 2π. An another complex unit vector satisfying $\bar{u}\cdot \bar{v}=0$ is given by

(15) $$\begin{array}{l} \bar{v}=\left(\begin{array}{c} \cos\chi \cos\theta \cos\phi e^{i\left(\beta_{1}-\alpha_{1}\right)}+\sin\chi \sin\phi e^{i\left(\beta_{2}-\alpha_{1}\right)}\\ \cos\chi \cos\theta \sin\phi e^{i\left(\beta_{1}-\alpha_{2}\right)}-\sin\chi \cos\phi e^{i\left(\beta_{2}-\alpha_{2}\right)}\\ -\cos\chi \sin\theta e^{i\left(\beta_{1}-\alpha_{3}\right)} \end{array}\right), \end{array}$$

where 0 ≤ χ ≤ π/2 and 0 ≤ β_1_, β_2_ ≤ 2π. The third complex unit vector $\bar{w}$ is determined from the orthogonality condition of the complex vectors. Then, a general SU(3) matrix is constructed by placing $\bar{u},{\bar{v}}^{*}$, and $\bar{w}$ as its columns (Mathur and Sen, 2001), and it can be written as

(16) $$\begin{array}{l} U=\left(\begin{array}{ccc} u_{1} & v_{1}^{*} & u_{2}^{*}v_{3}-v_{3}^{*}u_{2}\\ u_{2} & v_{2}^{*} & u_{3}^{*}v_{1}-v_{1}^{*}u_{3}\\ u_{3} & v_{3}^{*} & u_{1}^{*}v_{2}-v_{2}^{*}u_{1} \end{array}\right). \end{array}$$

Therefore, this 3 × 3 matrix is defined by eight real parameters ϕ, θ, χ, α_1_, α_2_, α_3_, β_1_, β_2_.

To start the game, we need to choose an initial state that is shared by the players. It can be assumed that an unbiased referee prepares the initial state and distributes the subsystems among the players. Thenceforth, no communication or interaction is allowed between the players and the referee. To choose an initial state, we need to fulfill three criteria: (a) The state should be entangled so that it can accommodate correlated randomization between the players. (b) The state must be symmetric and unbiased with respect to the positions of the players since the game follows these properties. (c) It must have the property of accessing the classical game through the restrictions on the strategy sets. A state that fulfills these criteria is given by

(17) $$\begin{array}{l} |\psi_{in}\rangle =\frac{1}{\sqrt{3}}(|000\rangle +|111\rangle +|222\rangle ). \end{array}$$

This is also a maximally entangled GHZ-type state that is defined on $H_{L}={\mathbb{C}}^{3}⊗{\mathbb{C}}^{3}⊗{\mathbb{C}}^{3}$. We consider this as the initial state to start the game.

To show that the assumed initial state satisfies the above criterion (c), we consider a set of operators representing the classical pure strategies that leads to deterministic payoffs when those are applied to the initial state |ψ_in_〉. This set of operators is given by the cyclic group of order 3, *C*_3_, generated by the 3 × 3 matrix

(18) $$\begin{array}{l} s=\;\left(\begin{array}{ccc} 0 & 0 & 1\\ 1 & 0 & 0\\ 0 & 1 & 0 \end{array}\right), \end{array}$$

with the following properties: *s*^0^ = *s*^3^ = *I* and *s*^2^ = *s*^−1^ = *s*^*T*^. Then the players choose their classical strategies from a set of operators *S* = {*s*^0^, *s*^1^, *s*^2^} with *s*^*a*^ ⊗ *s*^*b*^ ⊗ *s*^*c*^|000〉 = |*a b c*〉, where *a, b, c* ∈ {0, 1, 2}. By acting the set of classical strategies on the initial state |ψ_in_〉, we get the final state as

(19) $$\begin{array}{l} s^{a}⊗s^{b}⊗s^{c}\frac{1}{\sqrt{3}}(|000\rangle +|111\rangle +|222\rangle )\\ =\frac{1}{\sqrt{3}}(|0+a0+b0+c\rangle +|1+a1+b1+c\rangle \\ +|2+a2+b2+c\rangle ). \end{array}$$

It is important to note here, that the superscripts indicate the powers of the generator matrix and the addition is Modulo 3.

To proceed with the quantum game, an initial density matrix is constructed by using the initial state |ψ_in_〉 and adding a noise term, controlled by the parameter *f* (Schmid et al., 2010). The density matrix can be written as

(20) $$\begin{array}{l} \rho_{in}=f|\psi_{in}\rangle \langle \psi_{in}|+\frac{1-f}{27}I_{27}, \end{array}$$

where the parameter *f* ∈ [0, 1] and *I*_27_ is the 27 × 27 identity matrix. The parameter *f* is a measure of the fidelity of production of the initial state (Ramzan and Khan, 2012; Sharif and Heydari, 2012b). For *f* = 0, the initial state is fully random, since the corresponding density matrix has zero off-diagonal elements and non-zero diagonal elements are of equal strength. On the other hand, for *f* = 1, the initial state is entangled with zero noise. For the values of *f* between 0 and 1, the initial state is entangled with non-zero noise measured by *f*. Alice, Bob, and Charlie will now choose their strategies by considering a unitary operator *U*(ϕ, θ, χ, α_1_, α_2_, α_3_, β_1_, β_2_), and after their actions, the initial state ρ_*in*_ transforms into the final state

(21) $$\begin{array}{l} \rho_{fin}=U⊗U⊗U\rho_{in}U^{†}⊗U^{†}⊗U^{†}. \end{array}$$

Here we assume the same unitary operator *U* for all three players since there is no scope of communication among them. Therefore, it is practically impossible to coordinate which operator to be applied by whom. The next step is to construct a payoff operator *P*_*i*_ for each of the *i*-th player. This is defined as a sum of outer products of the basis states for which *i*-th player receives a payoff $ = 1. For example, the payoff operator of Alice is given by

(22) $$\begin{array}{l} P_{A}=(\overset{2}{\underset{y_{3},y_{2},y_{1}=0}{\sum }}|y_{3}y_{2}y_{1}\rangle \langle y_{3}y_{2}y_{1}|,y_{3}\neq y_{2},y_{3}\neq y_{1},y_{2}\neq y_{1})\\ \;+(\overset{2}{\underset{y_{3},y_{2},y_{1}=0}{\sum }}|y_{3}y_{2}y_{1}\rangle \langle y_{3}y_{2}y_{1}|,y_{3}=y_{2}\neq y_{1}). \end{array}$$

Note that the terms inside the first bracket of the operator represents the scenario when all three players have different choices, whereas the second bracket leads to the fact that Alice's choice is different from Bob and Charlie who have the same choices. In the same way, one can find the payoff matrices for Bob and Charlie. As defined in Equation (9), the expected payoff of player *i* can be calculated as

(23) $$\begin{array}{l} E_{i}\left(\$\right)=Tr\left(P_{i}\rho_{fin}\right), \end{array}$$

where *i* ∈ {*A, B, C*}.

The problem now is to find an optimal strategy, i.e., to determine a general unitary operator *U*(ϕ, θ, χ, α_1_, α_2_, α_3_, β_1_, β_2_) that maximizes the expected payoff. In this game, all the players will have the same expected payoff for a particular strategy operator, since they do not communicate with each other during the process. Therefore, the optimization of expected payoff can be done with respect to any of the three players. It has been shown in Sharif and Heydari (2011), that there exists an optimal unitary operator *U*^opt^ with the parameter values listed in Table 1, for which one finds a maximum expected payoff of *E*($) = 6/9, assuming a pure initial state [*f* = 1; refer to Equation (20)]. Thus, the quantum strategy outperforms classical randomization, and the expected payoff can be increased by 50% compared to the classical case where the expected payoff was found to be *E*_*c*_($) = 4/9. It also has been shown that *U*^opt^ is a Nash equilibrium, because no players can increase their payoff by changing their individual strategy from *U*^opt^ to any other strategy *U* (for details, refer to Sharif and Heydari, 2011).

**Table 1:** Parameter values for an optimal unitary operator *U*^opt^.

Parameter	*ϕ*	*θ*	*χ*	*α*_1_	*α*_2_	*α*_3_	*β*_1_	*β*_2_
Value	$\frac{\pi }{4}$	$\cos^{-1}\left(\frac{1}{\sqrt{3}}\right)$	$\frac{\pi }{4}$	$\frac{5\pi }{18}$	$\frac{5\pi }{18}$	$\frac{5\pi }{18}$	$\frac{\pi }{3}$	$\frac{11\pi }{6}$

By applying *U*^opt^ to the initial state (refer to Equation 17), the final state is given by

(24) $$\begin{array}{l} |\psi_{fin}\rangle =\frac{1}{3}(|000\rangle +|111\rangle +|222\rangle +|012\rangle +|021\rangle \\ \;+|102\rangle +|120\rangle +|201\rangle +|210\rangle ). \end{array}$$

Note that this is a collection of all the basis states that leads to providing a payoff either to all three players or none of them. We see that the optimal strategy profile *U*^opt^ ⊗ *U*^opt^ ⊗ *U*^opt^ becomes unable to get rid of the most undesired basis states |000〉, |111〉, |222〉 (i.e., no players will receive any payoff) from the final state. This failure is indeed responsible for getting expected payoff *E*($) = 6/9 instead of unity. For a general noise term *f* and optimal strategy, the expected payoff can be calculated as $E\left(\$\left(U^{\text{opt}},f\right)\right)=\frac{2}{9}\left(f+2\right)$ (Sharif and Heydari, 2011). This general result is compatible with the case of *f* = 1, and it also reproduces the classical value as *f* → 0.

### 4.1. Effect of Entanglement

We now investigate whether the level of entanglement of the initial state affects the payoffs of the players in quantum KPR problem with three players and three choices. To show this effect, one can start the game with a general entangled state

(25) $$\begin{array}{l} |\psi_{in}\rangle =\sin\vartheta \cos\varphi |000\rangle +\sin\vartheta \sin\varphi |111\rangle +\cos\vartheta |222\rangle , \end{array}$$

where 0 ≤ ϑ ≤ π and 0 ≤ φ ≤ 2π. Using the given optimal strategy *U*^opt^ and the above general initial state, the expected payoff can be found as

(26) $$\begin{array}{l} E\left(\$\left(U^{opt},\vartheta ,\varphi \right)\right)=\frac{1}{9}(\sin\left(\varphi \right)\sin\left(2\vartheta \right)+\cos\left(\varphi \right)(2\sin\left(\varphi \right)\sin^{2}\left(\vartheta \right)\\ \;+\sin\left(2\vartheta \right))+4). \end{array}$$

**Figure 3:**
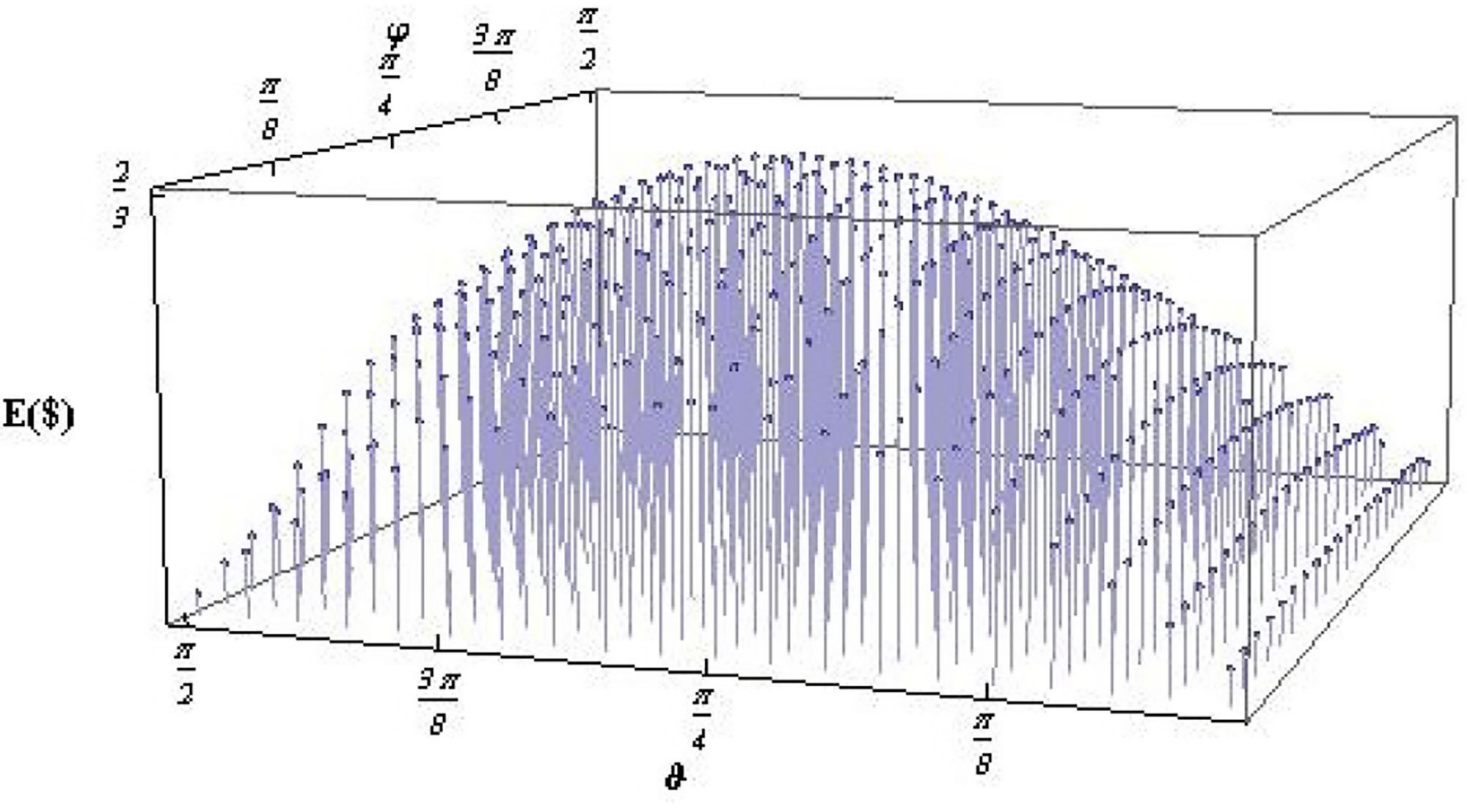
Expected payoff *E*($(*U*^opt^, ϑ, φ)) as a function of ϑ and φ, for a quantum KPR game with three players and three choices with the optimal strategy operator *U*^opt^. Each pair of ϑ and φ indicates a different initial state according to Equation (25). The peak in *E*($) occurs for a maximally entangled initial state, i.e., for $\vartheta =\cos^{-1}\left(1/\sqrt{3}\right)$ and φ = π/4 (taken from Sharif and Heydari, 2012c).

This relation is used to find the values of ϑ and φ for which the expected payoff becomes maximum. In Sharif and Heydari (2011, 2012a), it has been shown that the maximum expected payoff occurs for $\varphi =\frac{\pi }{4},\frac{3\pi }{4}$ and $\vartheta =\pm \cos^{-1}\left(1/\sqrt{3}\right)$, i.e., when the initial state is maximally entangled that we have considered in Equation (17). A small deviation from the maximal entangled state reduces the expected payoff from its maximum value (refer to Figure 3). It can be noted here that the expected payoff has a strong dependence on the level of entanglement of the initial state.

### 4.2. Effect of Decoherence

It is practically impossible to completely isolate a quantum system from the effects of the environment. Therefore, the studies that account for such effects have practical implications. In this context, the study of decoherence (or loss of phase information) is essential to understanding the dynamics of a system in presence of system-environment interactions. Quantum games are recently being explored to implement quantum information processing in physical systems (Pakuła, 2007) and can be used to study the effect of decoherence in such systems (Johnson, 2001; Chen et al., 2003; Flitney and Abbott, 2004; Ramzan and Khan, 2008, 2010). In this connection, different damping channels can be used as a theoretical framework to study the influence of decoherence in quantum game problems.

We here study the effect of decoherence in three-player and three-choice quantum KPR problem by assuming different noise models, such as amplitude damping, phase damping, depolarizing, phase flip and trit-phase flip channels, parameterized by a decoherence parameter *p*, where *p* ∈ [0, 1] (Ramzan, 2013). The lower limit of decoherence parameter represents a completely coherent system, whereas the upper limit represents the zero coherence or fully decohered case.

In a noisy environment, the Kraus operator representation can be used to describe the evolution of a quantum state by considering the super-operator Φ (Nielsen and Chuang, 2001). Using density matrix representation, the evolution of the state is given by

(27) $$\begin{array}{l} {\tilde{\rho }}_{f}=\Phi \left(\rho_{f}\right)=\underset{k}{\sum }E_{k}\rho_{f}E_{k}^{†}, \end{array}$$

where the Kraus operators *E*_*k*_ follow the completeness relation,

(28) $$\begin{array}{l} \underset{k}{\sum }E_{k}^{†}E_{k}=I. \end{array}$$

The Kraus operators for the game are constructed using the single qutrit Kraus operators as provided in Equations (30,31,32,33,34) by taking their tensor product over all *n*^3^ combination of π(*i*) indices

(29) $$\begin{array}{l} E_{k}=\underset{\pi }{⊗}e_{\pi \left(i\right)}, \end{array}$$

with *n* being the number of Kraus operators for a single qutrit channel. For the amplitude damping channel, the single qutrit Kraus operators are given by Ann and Jaeger (2009).

(30) $$\begin{array}{l} E_{0}=\left(\begin{array}{ccc} 1\; & 0\; & 0\\ 0\; & \sqrt{1-p}\; & 0\\ 0\; & 0\; & \sqrt{1-p} \end{array}\right),\;E_{1}=\left(\begin{array}{ccc} 0\; & \sqrt{p}\; & 0\\ 0\; & 0\; & 0\\ 0\; & 0\; & 0 \end{array}\right),\\ \;E_{2}=\left(\begin{array}{ccc} 0\; & 0\; & \sqrt{p}\\ 0\; & 0\; & 0\\ 0\; & 0\; & 0 \end{array}\right). \end{array}$$

In a similar way, the single qutrit Kraus operators for the phase damping channel can be found as (refer to e.g., Ramzan and Khan, 2012)

(31) $$\begin{array}{l} E_{0}=\sqrt{1-p}\left(\begin{array}{ccc} 1\; & 0\; & 0\\ 0\; & 1\; & 0\\ 0\; & 0\; & 1 \end{array}\right),\;E_{1}=\sqrt{p}\left(\begin{array}{ccc} 1\; & 0\; & 0\\ 0\; & \omega \; & 0\\ 0\; & 0\; & \omega^{2} \end{array}\right), \end{array}$$

where $\omega =e^{\frac{2\pi i}{3}}.$ For the depolarizing channel, the single qutrit Kraus operators take the forms as (refer to e.g., Salimi and Soltanzadeh, 2009),

(32) $$\begin{array}{l} E_{0}=\sqrt{1-p}I,\;E_{1}=\sqrt{\frac{p}{8}}Y,\;E_{2}=\sqrt{\frac{p}{8}}Z,\;E_{3}=\sqrt{\frac{p}{8}}Y^{2},\\ \;E_{4}=\sqrt{\frac{p}{8}}YZ, \end{array}$$

(33) $$\begin{array}{l} E_{5}=\sqrt{\frac{p}{8}}Y^{2}Z,\;E_{6}=\sqrt{\frac{p}{8}}YZ^{2},\;E_{7}=\sqrt{\frac{p}{8}}Y^{2}Z^{2},\;E_{8}=\sqrt{\frac{p}{8}}Z^{2}, \end{array}$$

where

(34) $$\begin{array}{l} Y=\left(\begin{array}{ccc} 0\; & 1\; & 0\\ 0\; & 0\; & 1\\ 1\; & 0\; & 0 \end{array}\right),\;Z=\left(\begin{array}{ccc} 1\; & 0\; & 0\\ 0\; & \omega \; & 0\\ 0\; & 0\; & \omega^{2} \end{array}\right). \end{array}$$

The single qutrit Kraus operators associated with the phase flip channel are given by

(35) $$\begin{array}{l} E_{0}=\left(\begin{array}{ccc} 1\; & 0\; & 0\\ 0\; & \sqrt{1-p}\; & 0\\ 0\; & 0\; & \sqrt{1-p} \end{array}\right),\;E_{1}=\left(\begin{array}{ccc} 0\; & \sqrt{p}\; & 0\\ 0\; & 0\; & 0\\ 0\; & 0\; & 0 \end{array}\right),\\ \;E_{2}=\left(\begin{array}{ccc} 0\; & 0\; & \sqrt{p}\\ 0\; & 0\; & 0\\ 0\; & 0\; & 0 \end{array}\right). \end{array}$$

Similarly, the single qutrit Kraus operators for the trit-phase flip channel can be found as

(36) $$\begin{array}{l} E_{0}=\sqrt{1-\frac{2p}{3}}\left(\begin{array}{ccc} 1\; & 0\; & 0\\ 0\; & 1\; & 0\\ 0\; & 0\; & 1 \end{array}\right),\;E_{1}=\sqrt{\frac{p}{3}}\left(\begin{array}{ccc} 0\; & 0\; & e^{\frac{2\pi i}{3}}\\ 1\; & 0\; & 0\\ 0\; & e^{\frac{-2\pi i}{3}}\; & 0 \end{array}\right),\\ E_{2}=\sqrt{\frac{p}{3}}\left(\begin{array}{ccc} 0\; & e^{\frac{-2\pi i}{3}}\; & 0\\ 0\; & 0\; & e^{\frac{2\pi i}{3}}\\ 1\; & 0\; & 0 \end{array}\right),\;E_{3}=\sqrt{\frac{p}{3}}\left(\begin{array}{ccc} 0\; & e^{\frac{2\pi i}{3}}\; & 0\\ 0\; & 0\; & e^{\frac{-2\pi i}{3}}\\ 1\; & 0\; & 0 \end{array}\right), \end{array}$$

where the term *p* = 1 − *e*^−Γ*t*^ determines the strength of quantum noise which is usually called a decoherence parameter. This relation describes the bounds [0, 1] of *p* by two extreme time limits *t* = 0, ∞, respectively. The final density matrix representing the state after the action of the channel is given by

(37) $$\begin{array}{l} \tilde{\rho_{f}}=\Phi_{p}\left(\rho_{f}\right) \end{array}$$

where Φ_*p*_ is the super-operator for realizing a quantum channel parameterized by the decoherence parameter *p*. The payoff operator for the *i*^th^ player (say Alice) is given by Equation (22). The expected payoff of *i*^th^ player can be calculated as

(38) $$\begin{array}{l} E_{i}\left(\$\right)=\text{Tr}\left\{P_{A}{\tilde{\rho }}_{f}\right\} \end{array}$$

where Tr represents the trace of the matrix. We have already studied the zero noise case (*p* = 0) in Section 4 considering the fidelity *f* = 1. It has been found that there exists an optimal unitary operator *U*_opt_ for which the expected payoff of a player becomes maximum. We here consider how a non-zero noise term *p* and the fidelity, *f* ≠ 1 affects the expected payoff.

**Figure 4:**
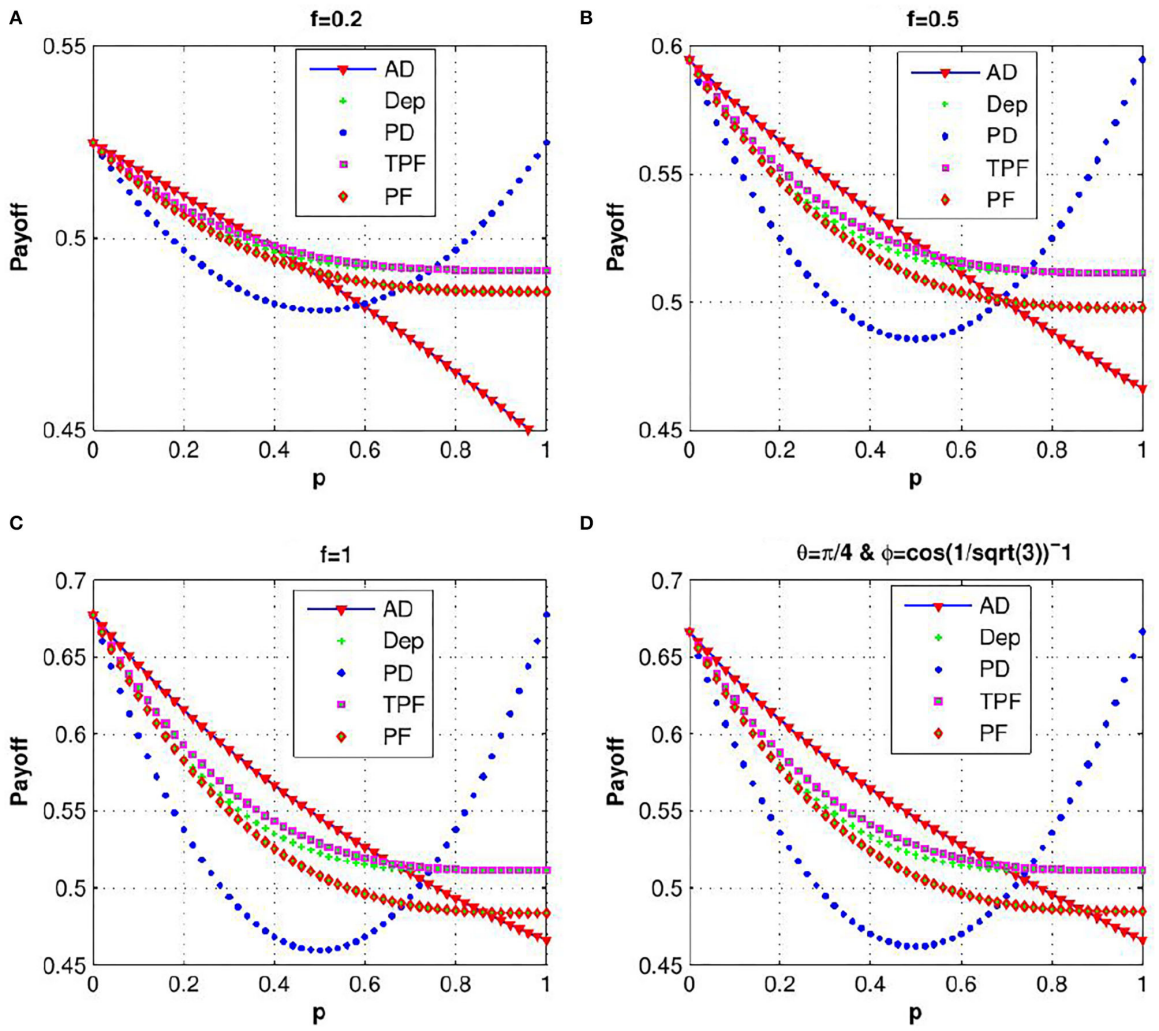
(Color online) Variation of Alice's expected payoff as a function of the decoherence parameter *p* for **(A)** *f* = 0.2, **(B)** *f* = 0.5, **(C)** *f* = 1, and **(D)** $\theta =\frac{\pi }{4},$ $\phi =\cos^{-1}\left(1/\sqrt{3}\right)$ for amplitude damping, depolarizing, phase damping, trit-phase flip, and phase flip, channels (taken from Ramzan, 2011).

**Figure 5:**
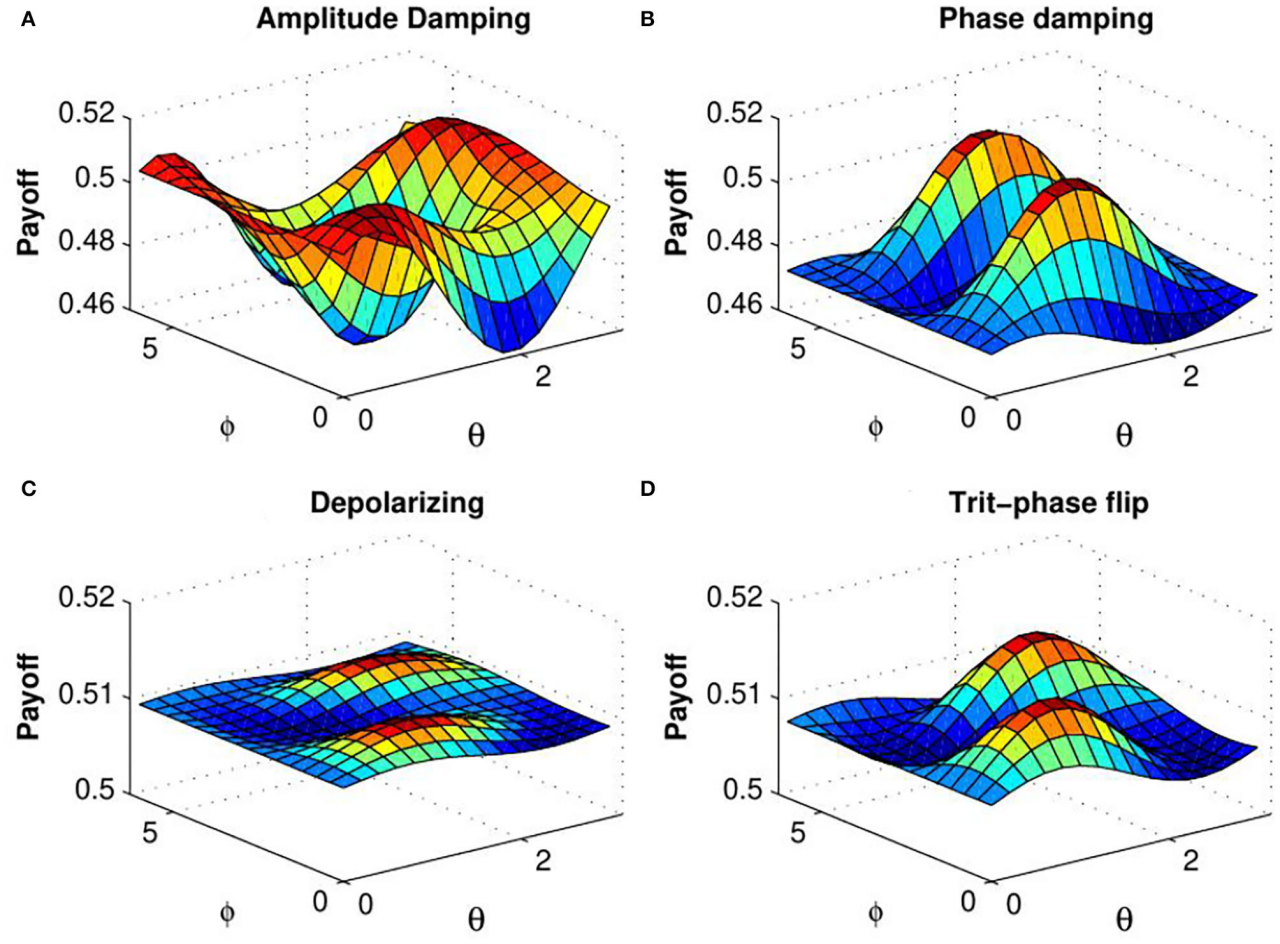
(Color online) Alice's expected payoff as a function of θ and ϕ [determined by Equation (25)] for **(A)** amplitude damping, **(B)** phase damping, **(C)** depolarizing, and **(D)** trit-phase flip channels with decoherence parameter *p* = 0.7 (taken from Ramzan, 2011).

**Figure 6:**
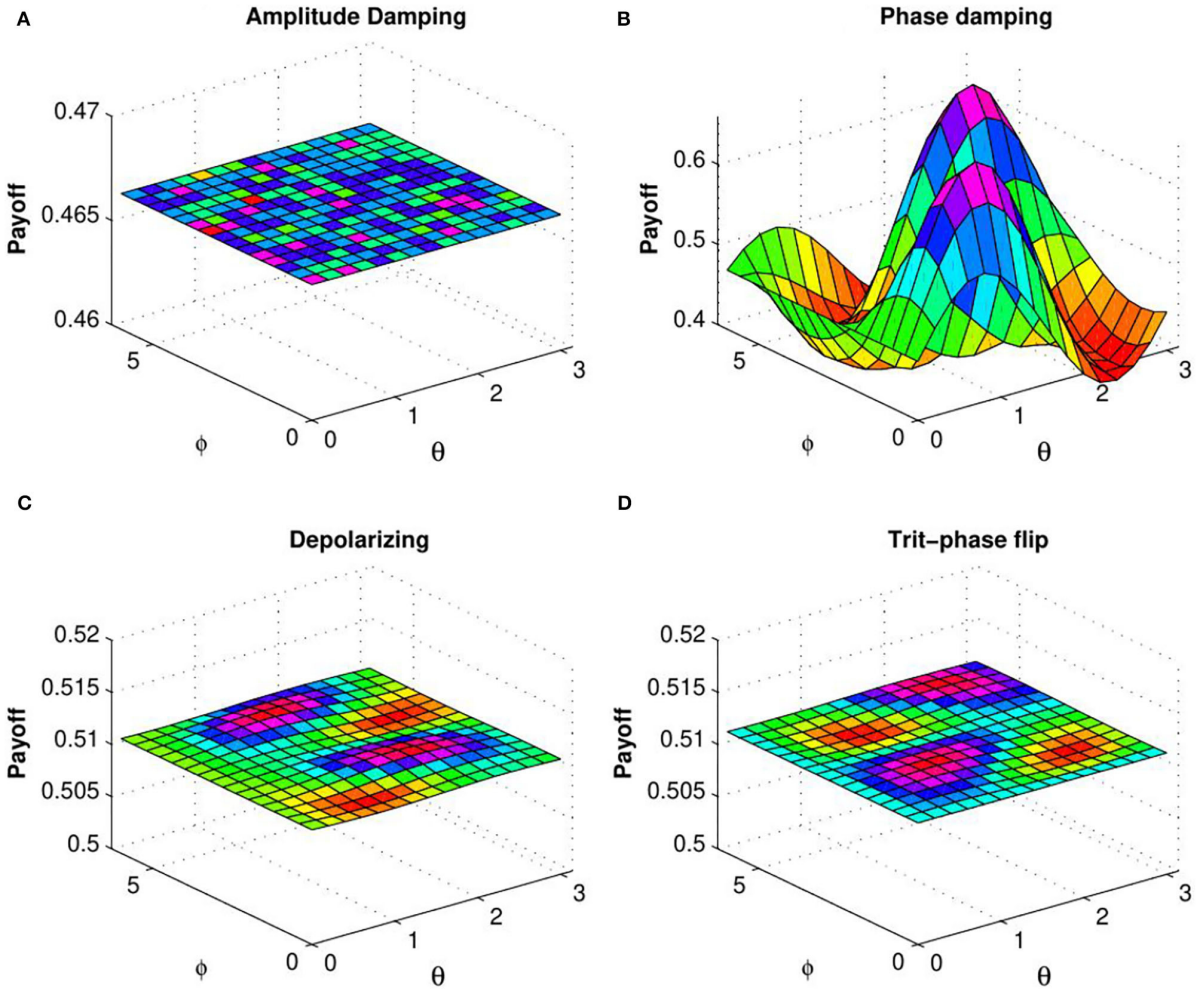
(Color online) Plot of Alice's expected payoff as a function of θ and ϕ [determined by Equation (25)] for **(A)** amplitude damping, **(B)** phase damping, **(C)** depolarizing, and **(D)** trit-phase flip channels with decoherence parameter *p* = 1 (from Ramzan, 2011).

In order to explain the effect of decoherence on the quantum KPR game, we investigate expected payoff by varying the decoherence parameter *p* for different damping channels. Due to the symmetry of the problem, we have considered expected payoff of one of the three players (say Alice) for further investigations. In Figure 4, the expected payoff of Alice is plotted as a function of decoherence parameter *p* for different values of fidelity *f* and different damping channels, such as amplitude damping, depolarizing, phase damping, trit-phase flip, and phase flip channels. It is observed that Alice's payoff is strongly affected by the amplitude driving channel as compared to the flipping and depolarizing channels. The effect of entanglement of the initial state is further investigated by plotting Alice's expected payoff as a function of θ and ϕ in presence of noisy environment with decoherence parameter *p* = 0.7 for different damping cases: (a) amplitude damping, (b) phase damping, (c) depolarizing, and (d) trit-phase flip channels (refer to Figure 5). In this scenario, one can see that Alice's payoff is heavily affected by depolarizing noise compared to the other noise cases. This plot is also repeated for the highest level of decoherence, i.e., *p* = 1 (see Figure 6). It is seen that there is a considerable amount of reduction in Alice's payoff for amplitude damping, depolarizing and trit-phase flip cases, whereas phase damping channel almost does not affect the payoff of Alice. Interestingly, the problem becomes noiseless for the maximum decoherence in the case of the phase damping channel. Finally, the maximum payoff is achieved for the case of the highest initial entanglement and zero noise, and it starts decreasing when the degree of entanglement deviates from maxima or introduces a non-zero decoherence term. Moreover, it has also been checked that the introduction of decoherence does not change the Nash equilibrium of the problem.

## 5. Summary and Discussion

In the Kolkata Paise Restaurant or KPR game λ*N* players choose every day independently but based on past experience or learning one of the *N* (→ ∞) restaurants in the city. As mentioned, the game becomes trivial if a non-playing dictator prescribes the moves to each player. Because of iterative learning, the KPR game is not necessarily a one-shot game, though it is so for random choice (no memory or learning from the past) by the players.

For random choices of restaurants by the players, the game effectively becomes one-shot with convergence time τ = 1 and steady utilization fraction *f* = 1 − *exp*(−λ) ≃ 0.63 (Chakrabarti et al., 2009), as shown through Equations 1, 2, 3 of Section 2. With iterative learning following Equations 5a,b for α = 1, λ = 1 it was shown numerically, as well as with a crude approximation in Ghosh et al. (2010b), that the utilization fraction *f* becomes of order 0.8 within a couple of iterations (τ of the order of 10). In Sinha and Chakrabarti (2020) the authors demonstrated numerically that for λ = 1, *f* can approach unity when α becomes 0_+_ from above. However, the convergence time τ at this critical point diverges due to critical slowing down (refer to e.g., Stanley, 1987), rendering such critically slow leaning of full utilization is hard to employ for practical purposes (San Miguel and Toral, 2020). The cases of λ < 1 (and α = 1) were considered earlier in Ghosh et al. (2012) and Sinha and Chakrabarti (2020), and have also been studied here in Section 2, using the Monte Carlo technique. A mean field-like transition (refer to e.g., Stanley, 1987) is observed here giving full utilization (*f* = 1) for λ less than λ_*c*_ about 0.75 where τ also remains finite. As shown in Figure 1, τ diverges at the critical point λ_*c*_ (a crude mean field derivation of it is given in Equation 6).

For the quantum version of the KPR problem, we have discussed the one-shot game with three players and three choices that was first introduced in Sharif and Heydari (2011); Also refer to Sharif and Heydari (2012b). For this particular KPR game with three players and three choices, the classical randomization provides a total of 27 possible configurations, and 12 out of them gives a payoff $ = 1 to each of the players, thus leading to an expected payoff *E*_*c*_($) = 4/9. On the other hand, for the quantum case, it has been shown that when the players share a maximally entangled initial state, there exists a local unitary operation (same for all the players due to the symmetry of the problem) for which the players receive a maximum expected payoff *E*_*q*_($) = 6/9, i.e., the quantum players can increase their expected payoff by 50% compared to their classical counterpart. To show the effect of entanglement, the expected payoff is calculated for a general GHZ-type initial state with different levels of entanglement (refer to Figure 3). It appears that the maximally entangled initial state provides maximum payoff *E*_*q*_($) = 6/9 to each of the players. The expected payoff decreases from its maximum value for any deviation from the maximum entanglement of the initial state (refer to Figure 3). This is the highest expected payoff that is attained so far for a one-shot quantum KPR game with three players and three choices (refer to Sharif and Heydari, 2012a). Until now, the study of the quantum KPR problem is limited to one shot with three players and three choices, and no attempt is found yet to make it iterative, and also to increase the number of players and choices. Therefore, it is yet to be understood whether one can increase the expected payoff by making the quantum KPR game iterative with learning from previous rounds as happened in the case of classical strategies studied here. As a consequence, unlike the classical KPR case, the development of the quantum version of the KPR game is at a preliminary stage, and its practical applications are not explored yet. However, the applications of quantum game theory have recently been made, with some success, to simulate recovery in a mobile database system (refer to e.g., Madbouly et al., 2021).

Decoherence is an unavoidable phenomenon for quantum systems since it is not possible to completely isolate a system from the effects of the environment. Therefore, it is important to investigate the influence of decoherence on the payoffs of the players in the context of quantum games. The effect of decoherence in a three-player and three-choice quantum KPR problem is studied in Ramzan (2013) using different noise models like amplitude damping, phase damping, depolarizing, phase flip, and trit-phase flip channels, parametrized by a decoherence parameter. The lower and upper limits of the decoherence parameter represent the fully coherent and fully decohered system, respectively. Expected payoff is reported to be strongly affected by amplitude damping channel as compared to the flipping and depolarizing channels for the lower level of decoherence, whereas it is heavily influenced by depolarizing noise in case of a higher level of decoherence. However, for the case of the highest level of decoherence, amplitude damping channel dominates over the depolarizing and flipping channels, and the phase damping channel has nearly no effect on the payoff. Importantly, the Nash equilibrium of the problem is shown not to be changed under decoherence.

There have been several applications of KPR game strategies to various social optimization cases. KPR game has been extended to Vehicle for Hire Problem in Martin (2017) and Martin and Karaenke (2017). Authors have built several model variants such as Individual Preferences, Mixed Preferences, Individual Preferences with Multiple Customers per District, and Mixed Preferences and Multiple Customers per District. Using these variants, authors have studied various strategies for the KPR problem that led to the foundation of an incentive scheme for dynamic matching in mobility markets. Also in Martin and Karaenke (2017), a modest level of randomization in choice along with mixed strategies is shown to achieve around 80% of efficiency in the vehicle for hire markets. A time-varying location specific resource allocation crowd-sourced transportation is studied using the methodology of mean field equilibrium (Yang et al., 2018). This study provides a detailed mean field analysis of the KPR game and also considers the implications of an additional reward function. In Park and Saad (2017), a resource allocation problem for a large IoT system, consisting of IoT devices with imperfect knowledge, is formulated using the KPR game strategies. The solution, where those IoT devices autonomously learn equilibrium strategies to optimize their transmission, is shown to coincide with the Nash equilibrium. Also, several ‘emergent properties’ of the KPR game, such as the utilization fraction or the occupation density in the steady or stable states, and phase transition behavior, have been numerically studied in Tamir (2018). A search problem often arises as a time-limited business opportunity by business firms, and is studied like a one-shot delegated search in Hassin (2021). Authors here had discussed and investigated the searchers' incentives following different strategies, including KPR, can maximize search success. Authors in Kastampolidou et al. (2021) have discussed KPR problem as Traveling Salesman Problem (TSP) assuming restaurants are uniformly distributed on a two-dimensional plane and this topological layout of the restaurants can help provide each agent a second chance for choosing a better or less crowded restaurant. Additionally, they have proposed a meta-heuristics, producing near-optimal solutions in finite time (as exact solutions of the TSP are prohibitively expensive). Thus, agents are shown to learn fast, even incorporating their own preferences, and achieve maximum social utilization in lesser time with multiple chances.

## Data Availability Statement

The raw data supporting the conclusions of this article will be made available by the authors, without undue reservation.

## Author Contributions

Planning and designing by BC. Classical data by AS. Quantum study by AR. All authors contributed in data analysis, writing, and editing the paper.

## Conflict of Interest

The authors declare that the research was conducted in the absence of any commercial or financial relationships that could be construed as a potential conflict of interest.

## Publisher's Note

All claims expressed in this article are solely those of the authors and do not necessarily represent those of their affiliated organizations, or those of the publisher, the editors and the reviewers. Any product that may be evaluated in this article, or claim that may be made by its manufacturer, is not guaranteed or endorsed by the publisher.

## References

[B1] Ann K. Jaeger G. (2009). Finite-time destruction of entanglement and non-locality by environmental influences. Foundat. Phys. 39, 790. 10.1007/s10701-009-9295-8

[B2] Benjamin S. C. Hayden P. M. (2001). Multiplayer quantum games. Phys. Rev. A 64, 030301. 10.1103/PhysRevA.64.030301 11497872

[B3] Bleiler S. A. (2008). A formalism for quantum games and an application. arXiv:0808.1389. 10.48550/arXiv.0808.1389

[B4] Chakrabarti A. S. Chakrabarti B. K. Chatterjee A Mitra M. (2009). The Kolkata Paise Restaurant problem and resource utilization. Physica A 388, 2420. 10.1016/j.physa.2009.02.039

[B5] Chakrabarti B. K. (2007). Kolkata restaurant problem as a generalised el farol bar problem, in Econophysics of Markets and Business Networks (Springer), 239–246.

[B6] Chakrabarti B. K. Chatterjee A. Ghosh A. Tamir B. (2017). Econophysics of the Kolkata Restaurant Problem and Related Games: Classical and Quantum Strategies for Multi-agent, Multi-choice Repetitive Games. New Economic Windows; Cham: Springer.

[B7] Chakrabarti B. K. Sinha A. (2021). Development of econophysics: a biased account and perspective from Kolkata. Entropy 23, 254. 10.3390/e23020254 33672245 PMC7926551

[B8] Challet D. Marsili M. Zhang Y-. C. (2005). Minority Games: Interacting Agents in Financial Markets. Oxford: Oxford University Press.

[B9] Chen L. Ang H. Kiang D. Kwek L. Lo C. (2003). Quantum prisoner dilemma under decoherence. Phys. Lett. A 316, 317. 10.1016/S0375-9601(03)01175-7

[B10] Chen Q. Wang Y. Liu J-.T. Wang K. (2004). N-player quantum minority game. Phys. Lett. A 327, 98. 10.1016/j.physleta.2004.05.012

[B11] Eisert J. Wilkens M. Lewenstein M. (1999). Quantum games and quantum strategies. Phys. Rev. Lett. 83, 3077. 10.1103/PhysRevLett.83.3077

[B12] Flitney A. P. Abbott D. (2004). Quantum games with decoherence. J. Phys. A Math. Gen. 38, 449. 10.1088/0305-4470/38/2/01121554890

[B13] Ghosh A. Chakrabarti A. S. Chakrabarti B. K. (2010a). Kolkata Paise Restaurant problem in some uniform learning strategy limits, in Econophysics and Economics of Games, Social Choices and Quantitative Techniques (Heidelberg: Springer), 3–9.

[B14] Ghosh A. Chatterjee A. Mitra M. Chakrabarti B. K. (2010b). Statistics of the kolkata paise restaurant problem. New J. Phys. 12, 075033. 10.1088/1367-2630/12/7/075033

[B15] Ghosh A. Martino D. D. Chatterjee A. Marsili M. Chakrabarti B. K. (2012). Phase transitions in crowd dynamics of resource allocation. Phys. Rev. E 85, 021116. 10.1103/PhysRevE.85.021116 22463162

[B16] Hassin R. (2021). Equilibrium and optimal one-shot delegated search. IISE Trans. 53, 928. 10.1080/24725854.2019.1663085

[B17] Johnson N. F. (2001). Playing a quantum game with a corrupted source. Phys. Rev. A 63, 020302. 10.1103/PhysRevA.63.020302

[B18] Kastampolidou K. Papalitsas C. Andronikos T. (2021). DKPRG or how to succeed in the kolkata paise restaurant game via TSP. arXiv:2101.07760. 10.48550/arXiv.2101.07760

[B19] Landsburg S. E. (2011). arXiv, 1110.6237. Available online at: https://arxiv.org/pdf/1110.6237v1.pdf.

[B20] Lockwood B. (2008). Pareto Efficiency, The New Palgrave Dictionary of Economics, 2nd Edn. London, UK: Palgram Macmillan.

[B21] Madbouly M. M. Mokhtar Y. F. Darwish S. M. (2021). Quantum game application to recovery problem in mobile database. Symmetry 13, 1984. 10.3390/sym13111984

[B22] Marinatto L. Weber T. (2000). A quantum approach to static games of complete information. Phys. Lett. A 272, 291. 10.1016/S0375-9601(00)00441-2

[B23] Martin L. (2017). Extending Kolkata Paise Restaurant problem to dynamic matching in mobility markets. Junior Manag. Sci. 4, 1. 10.5282/jums/v4i1pp1-34

[B24] Martin L. Karaenke P. (2017). The vehicle for hire problem: a generalized Kolkata Paise Restaurant problem, in Workshop on Information Technology and Systems. Available online at: https://mediatum.ub.tum.de/doc/1437330/1437330.pdf.

[B25] Mathur M. Sen D. (2001). Coherent states for SU (3). J. Math. Phys. 42, 4181. 10.1063/1.1385563

[B26] Meyer D. A. (1999). Quantum strategies. Phys. Rev. Lett. 82, 1052. 10.1103/PhysRevLett.82.1052

[B27] Morgenstern O. Von Neumann J. (1953). Theory of Games and Economic Behavior. Princeton: Princeton University Press.

[B28] Nielsen M. A. Chuang I. L. (2001). Quantum computation and quantum information. Phys. Today 54, 60. 10.5555/1972505

[B29] Osborne M. J. Rubinstein A. (1994). A Course in Game Theory. Massachusetts: MIT Press.

[B30] Pakuł I. (2007). Quantum gambling using mesoscopic ring qubits. Physica Status Solidi 244, 2513. 10.48550/arXiv.quant-ph/0610037

[B31] Park T. Saad W. (2017). Kolkata paise restaurant game for resource allocation in the internet of things, in 2017 51st Asilomar Conference on Signals, Systems, and Computers (Pacific Grove, CA: IEEE), 1774–1778.

[B32] Piotrowski E. W. Sładkowski J. (2003). An invitation to quantum game theory. Int. J. Theor. Phys. 42, 1089. 10.1023/A:1025443111388

[B33] Prisoner's Dilemma. (2019). The Stanford Encyclopedia of Philosophy. Available online at: https://plato.stanford.edu/entries/prisoner-dilemma/.

[B34] Ramzan M. (2011). arXiv, 1111.3913. Available online at: https://arxiv.org/pdf/1111.3913.pdf.

[B35] Ramzan M. (2013). Three-player quantum Kolkata restaurant problem under decoherence. Quantum Inform. Process. 12, 577. 10.1007/s11128-012-0405-8

[B36] Ramzan M. Khan M. (2008). Noise effects in a three-player prisoner's dilemma quantum game. J. Phys. A 41, 435302. 10.1088/1751-8113/41/43/435302

[B37] Ramzan M. Khan M. (2010). Distinguishing quantum channels via magic squares game. Quantum Inform. Process. 9, 667. 10.1007/s11128-009-0155-4

[B38] Ramzan M. Khan M. (2012). Decoherence and entanglement degradation of a qubit-qutrit system in non-inertial frames. Quantum Inform. Process. 11, 443. 10.1007/s11128-011-0257-7

[B39] Salimi S. Soltanzadeh M. (2009). Investigation of quantum roulette. Int. J. Quantum Inform. 7, 615. 10.1142/S0219749909004992

[B40] San Miguel M. Toral R. (2020). Introduction to the chaos focus issue on the dynamics of social systems. Chaos 30, 120401. 10.1063/5.0037137 33380029

[B41] Schmid C. Flitney A. P. Wieczorek W. Kiesel N. Weinfurter H. Hollenberg L. (2010). Experimental implementation of a four-player quantum game. New J. Phys. 12, 063031. 10.1088/1367-2630/12/6/063031

[B42] Sharif P. Heydari H. (2011). Quantum solution to a three player Kolkata restaurant problem using entangled qutrits. arXiv:1111.1962. New York, NY: Ithaca. 10.48550/arXiv.1111.1962

[B43] Sharif P. Heydari H. (2012a). Strategies in a symmetric quantum Kolkata restaurant problem. AIP Conf. Proc. 1508, 492–496. 10.1063/1.4773171

[B44] Sharif P. Heydari H. (2012b). An introduction to multi-player, multi-choice quantum games. arXiv:1204.0661. 10.48550/arXiv.1204.0661

[B45] Sharif P. Heydari H. (2012c). arXiv, 1212.6727. Available online at: https://arxiv.org/pdf/:1212.6727.

[B46] Sharif P. Heydari H. (2013). An introduction to multi-player, multi-choice quantum games: quantum minority games and kolkata restaurant problems, in Econophysics of Systemic Risk and Network Dynamics (Springer), 217–236.

[B47] Sinha A. Chakrabarti B. K. (2020). Phase transition in the Kolkata Paise Restaurant problem. Chaos 30, 083116. 10.1063/5.0004816 32872841

[B48] Stanley H. E. (1987). Introduction to Phase Transitions and Critical Phenomena. Oxford: Oxford University Press.

[B49] Tamir B. (2018). Econophysics and the Kolkata Paise Restaurant Problem: more is different. Sci. Cult. 84, 37. https://www.scienceandculture-isna.org/jan-feb-2018/05%20Art_Econophysics_and_the_Kolkata_Paise…by_Boaz%20Tamir_Pg.37.pdf

[B50] Yang P. Iyer K. Frazier P. (2018). Mean field equilibria for resource competition in spatial settings. Stochastic Syst. 8, 307. 10.1287/stsy.2018.0018

